# The Pattern of Stability and Change in Parental Locus of Control Over 6 Years and Teacher Ratings of Child Behavior

**DOI:** 10.3389/fpsyg.2018.01427

**Published:** 2018-08-08

**Authors:** Stephen Nowicki, Steven Gregory, Genette L. Ellis, Yasmin Iles-Caven, Jean Golding

**Affiliations:** ^1^Department of Psychology, Emory University, Atlanta, GA, United States; ^2^Centre for Academic Child Health, Bristol Medical School, University of Bristol, Bristol, United Kingdom

**Keywords:** ALSPAC, parental locus of control, child behavior, teacher SDQ, change over time, mother, father, longitudinal cohort study

## Abstract

A previous study from our group showed that parents’ locus of control (LOC) obtained before the birth of their child was associated with the child’s behavior at school in School Years 3 (ages 7–8) and 6 (ages 10–11). Here we examine whether a change in parental LOC over the first 6 years of the child’s life was associated with differences in his or her behavior as rated by their teachers. As before, we use data from the Avon Longitudinal Study of Parents and Children (ALSPAC). A modified version of the adult Nowicki–Strickland internal–external LOC scale was completed by mothers and fathers in their own home during pregnancy and 6 years later. Externality was defined as a score greater than the median and internality as equal to, or less than, the median. Outcomes were the five individual subscales and the total difficulties of Goodman’s Strengths and Difficulties Questionnaire (SDQ) completed by the children’s class teachers at the end of School Years 3 and 6. As predicted, we found that parents who remained externally oriented, or became external, had children with more behavioral difficulties in primary school compared with parents who remained or became internal. Type of behavior difficulties varied somewhat with whether mothers or fathers remained or changed toward externality. These results support the possibility that changes in parental LOC are associated with children’s personal and social adjustment. Consequently, programs to change parental LOC may be worth evaluating.

## Introduction

Locus of control (LOC) is a personality construct related to a variety of outcomes. Rotter (1966) introduced that construct and defined it as follows: “Internal versus external control refers to the degree to which persons expect that a reinforcement or an outcome of their behavior is contingent on their own behavior or personal characteristics versus the degree to which persons expect that the reinforcement or outcome is a function of chance, luck, or fate, is under the control of powerful others, or is simply unpredictable. Such expectancies may generalize along a gradient based on the degree of semantic similarity of the situational cues.”

Investigators have published thousands of LOC studies using adults (Nowicki and Duke, 2016) and found internality (having an internal LOC) related to *higher academic achievement* (e.g., Kalechstein and Nowicki, 1997; Flouri, 2006), *better sports performance* (e.g., Arnaud and Palazzolo, 2012), and *business success* (e.g., Spector et al., 2002; Wu et al., 2015); and externality (having an external LOC) to a variety of *negative personality characteristics* (e.g., Duke and Nowicki, 1973; Wheeler and White, 1991), *depression* (e.g., Bjørkløf et al., 2013), *anxiety* (Carden et al., 2004), and *psychoses* (Harrow et al., 2009; Weintraub et al., 2016).

External LOC is also associated with negative personal and social outcomes in children (Nowicki and Duke, 1983; Nowicki, 2016, unpublished), *suicidal* (Liu et al., 2005), *depressed* (Benassi et al., 1988; Luthar and Blatt, 1993), *enuretic* (Butler, 2001), *learning disabled* (Dudley-Marling et al., 1982), having *lower self-esteem* (Wickline et al., 2011), *attention deficit disorder* (Ialongo et al., 1993), and *less ability to persist* (McLeod, 1985).

While external orientation has been related to negative outcomes in both adults and children, few researchers have examined the possible associative role parent externality might play in their children’s lives. There are both theoretical and empirical reasons for assuming parent LOC might be associated with child outcomes. Descriptively, internality is characterized by factors that should make for better parenting, e.g., being more thoughtful, less impulsive, more responsible, persistent and able to delay gratification, as well as tendencies to gather more information from environments (Rotter, 1966, 1975, 1990; Lefcourt, 1976, 1981, 1982, 1983). Campis et al. (1986) suggest that parents “with external parental LOC orientations possess several negative concomitant attitudes about their parental roles such as low self-efficacy and a sense of being dominated by their child’s demands.” (p. 265). Lefcourt (1982) agrees and reasoned not only that externally controlled parents would tend to see their children’s behavior as being outside of their efforts, but they also would be inconsistent in setting limits for their children’s activities. Lack of structure, consistency, and limits would create problems for children attempting to learn, through feedback, how to behave appropriately. Empirically, parental internality has been associated with positive, and externality with negative child outcomes (Nowicki, 2016). Using a specific parenting LOC scale (Campis et al., 1986), researchers found that parenting externality in one or both parents was associated with negative outcomes in *preschool* (e.g., Estroff et al., 1994), *preadolescent, and adolescent participants* (e.g., Freed and Tompson, 2011) as well as a greater likelihood of receiving diagnoses of *Attention Deficit/Hyperactivity* (Hoza et al., 2000) or *anxiety* (Becker et al., 2010).

The possible connection between the parenting LOC and children’s outcomes is supported by others. For example, Hagekull et al. (2001) found that greater parenting externality, measured when children were aged 33 months and 9 years, was related to greater child difficulties both concurrently and prospectively. They concluded that parents’ perceived control is important for their children’s development of externalizing and internalizing problems as well as for social and non-social competence development, and of having an independent impact on development during the preschool years over and above infant temperament and acting out behavior.

Using a generalized LOC scale consistent with Rotter’s definition (i.e., one that is not focused on particular features such as a medical condition or parenting strategies), Nowicki et al. (2017) found an association between prenatal parent LOC and children’s personality and social behavior. They used data from the Avon Longitudinal Study of Parents and Children (ALSPAC) (Golding and ALSPAC Study Team, 2004; Boyd et al., 2013; Fraser et al., 2013), reporting that prenatal parent externality was associated with greater numbers of child eating, sleeping, and anger management problems during the first 5 years.

To evaluate whether the association of prenatal parent externality with negative children’s outcomes extended into later childhood, Nowicki et al. (2018b) compared teacher ratings of students’ emotion and behavior difficulties using the Strengths and Difficulties Questionnaire (SDQ) (Goodman, 1997, 2001) in School Years 3 and 6. Generally, prenatal parent externality was positively associated with a greater number of difficulties. Father externality was associated strongly with conduct difficulties for boys and mother externality with emotion problems for girls, but total number of teacher rated difficulties was related to the presence of externality in both parents. These findings were consistent with those of Hagekull et al. (2001) who found that mothers’ externality was more strongly associated with children’s internalizing difficulties in contrast to fathers’ externality which was related to externalizing problems.

For the present study, we sought to examine how stability and change in parent LOC over the first 6 years of children’s lives were associated with their emotional and behavioral difficulties at the end of School Years 3 and 6. Previously, Nowicki et al. (2018a) found a correlation of 0.55–0.56 between each parent’s LOC before the birth of their child and 6 years later. These moderate correlations suggest that many parents changed their LOC orientation over the 6 years. Our aim was to evaluate whether the degree and type of parent change in LOC over time was differentially associated with children’s emotional and behavior difficulties.

Previous results showed parental externality as measured by specific parenting LOC (e.g., Campis et al., 1986) or generalized LOC (Golding et al., 2017; Nowicki et al., 2018b), was related to negative child outcomes. Based on past empirical results, we predicted that children of parents who remained external or changed from internal to external would have a greater number of behavior difficulties when compared to parents who remained, or became, internal during that time.

## Materials and Methods

### The Population Studied

This research takes advantage of data collected by ALSPAC. Initiated in 1990, it recruited 14,541 pregnant women who were resident in an area of south-west England, and due to deliver between 1st April 1991 and 31st December 1992. The initial number of pregnancies enrolled is 14,541 (for these at least one questionnaire has been returned or a “Children in Focus” clinic had been attended by 19/07/99). Of these initial pregnancies, there was a total of 14,676 fetuses, resulting in 14,062 live births and 13,988 children who were alive at 1 year of age. The aim was to study ways in which the environment (including psychological, social as well as chemical impacts) together with the genetic characteristics, influenced the health, development and well-being of the offspring. Ethical approval for the study was obtained from the ALSPAC Ethics and Law Committee and the local research ethics committees (Birmingham, 2018). The ALSPAC Ethics and Law Advisory Committee agreed that consent was implied if questionnaires were returned. Informed written consent was obtained for all biological samples prior to analysis, and for certain invasive procedures during the hands-on assessments (which were optional to attend).

Data were collected using a variety of resources including self-completion questionnaires (completed by parents, children, and teachers), assays of biological samples, linkage to other data sets, and hands-on examinations. For this study, we use data from parents in pregnancy and 6 years later, linked to data from primary school teachers of their offspring at two time points [School Years 3 and 6 (aged 7–8 and 10–11), respectively]. Please note that the study website contains details of all the data that is available through a fully searchable data dictionary and variable search tool: http://www.bristol.ac.uk/alspac/researchers/our-data/

### The Exposures: Parental LOC

ALSPAC used the Adult Nowicki Strickland Internal External control scale (ANSIE) (Nowicki and Duke, 1974, 1983; Nowicki, 2016, unpublished) that followed Rotter’s definition in its construction. It has an easier reading level than the Rotter scale and is significantly correlated with Rotter’s test (Nowicki and Duke, 1983) making it appropriate for testing adults from the general population. The scale has been used in over 2,000 studies (Nowicki, 2016, unpublished).

More specifically, Nowicki (2016) reports split-half reliabilities in the 0.60 s for college (*n* = 156) and community samples (*n* = 33). These split-half reliabilities seem to be satisfactory in light of the fact that these personality items are not arranged according to difficulty. This makes the split-half reliabilities an underestimate of the true internal consistency reliability. Further report of internal consistency estimates of KR20s of 0.69 for a male sample (*n* = 40) and 0.39 for female sample (*n* = 40) were found. Nowicki and Duke (1983) reported test–retest reliability for college subjects over a 6-week period were 0.83 (*n* = 48), which was comparable to what Chandler (1976) found over a 7-week period (*r* = 0.65, *n* = 70) and Roueche and Mink (1976) found over a year (*r* = 0.56, *n* = 854) for community college students.

An anglicized and briefer form of the ANSIE was administered to each of the parents in pregnancy and 6 years later. It contained 12 items from the original 40-item scale which possessed the highest item-total correlations based on the responses of a pilot sample of 135 mothers. Each item had a simple binary answer, with the consequence that the score ranged from 0 to 12. The scales were completed by each parent at home. The higher the score the more external the LOC. As in our previous analyses, external LOC was defined as above the median while internal LOC was defined as scores equal to, or lower than, the median. The median score for the mother at the two-time points was 4 and 3, and for the father it was 3 at both.

### The Outcome Measure: Child’s Behavior

The behavior of the study child was recorded on paper questionnaires, one for each child in the class, by the child’s class teachers toward the end of School Years 3 and 6. All primary schools in the study area were approached, and for the children who had moved out of the area, parents were sent the questionnaire with a reply paid envelope to give to the teacher.

The child’s behavior was rated using the 25-item SDQ (Goodman, 1997), a well-validated method that measures five attributes: prosocial behavior, hyperactivity, conduct problems, emotional difficulties, and peer problems. Each attribute comprises the answers to five questions rated on a three-point Likert scale (0 – not true; 1 – somewhat true; or 2 – certainly true). From these questions scores were calculated, the higher the score the more problematic the behavior, except for the prosocial score where the reverse was true. If the answer to an item was missing, the score was prorated taking account of the other answers in that subscale. A ‘total difficulties score’ is the sum of the behaviors (excluding the prosocial behavior) (Goodman, 1997). Internal consistency across the different constructs of the SDQ and across different informants (self-report, teacher, and parent) has been found to be satisfactory (Cronbach’s Alpha mean of 0.73). Test–retest stability after 4–6 months has been reported to be 0.62 (Goodman, 2001).

### Data Analysis

We documented ways in which changes in LOC of each parent were associated with the child’s behavior as reported by the teacher. This analysis assesses whether mothers’ and fathers’ changes in LOC orientations have similar or different associations and stratifies by sex of the child to determine whether there are different associations. Numbers in each of the strata are found in the last rows of the tables.

For measuring change over time, we considered four strata for each parent: those that stayed external at each time point (Ext.Ext); those who started external but became internal (Ext.Int); those who were internal at both time points (Int.Int), and those who started internal, but were external 6 years later (Int.Ext). Results are given as the mean behavior score with its standard deviation (SD). As in a previous paper (Nowicki et al., 2017), we compare statistically the outcomes between (Ext.Ext) and (Ext.Int), as well as between (Int.Ext) and (Int.Int), using a two-tailed 2-sample *t*-test.

As a supplementary exercise (**Supplementary Tables 5, 6**), we present the effect sizes of differences in behavior scores using Cohen’s *d* (Valentine and Cooper, 2003), and employing the standard deviation of the reference group (i.e., those who remained internal).

## Results

### Comparison of the Behaviors Over Time

Comparison of the behavior scores in years 3 and 6 indicates that, in general, the children became more prosocial, less hyperactive and had fewer total difficulties within each of the maternal change groups, whereas the other types of behavior were not consistent in this respect (**Table 1**).

**Table 1:** Relationship between the mean [*SD*] scores of child’s behavior using the Strengths and Difficulties Questionnaire (SDQ) and the **changes in maternal LOC** between pregnancy and 6 years later.

Outcome and school year	Stayed external	Changed external to internal	Changed internal to external	Stayed internal
*Prosocial*				
Year 3	7.68 [2.43]	7.68 [2.43]	7.79 [2.40]	8.02 [2.30]^d^
Year 6	7.95 [2.39]	7.95 [2.39]	7.94 [2.38]	8.09 [2.26]
*Hyperactivity*				
Year 3	2.75 [2.68]	2.65 [2.72]	2.49 [2.66]	2.04 [2.34]^c^
Year 6	2.39 [2.67]	1.92 [2.44]^a^	2.06 [2.50]	1.75 [2.33]^c^
*Emotional*				
Year 3	1.43 [1.97]	1.38 [2.02]	1.26 [1.83]	1.18 [1.76]
Year 6	1.30 [1.88]	1.16 [1.82]	1.25 [1.84]	1.12 [1.67]^d^
*Conduct Problems*				
Year 3	0.77 [1.47]	0.74 [1.45]	0.63 [1.27]	0.48 [1.06]^d^
Year 6	0.86 [1.57]	0.61 [1.20]^b^	0.68 [1.37]	0.55 [1.19]^d^
*Peer Difficulties*				
Year 3	1.23 [1.82]	1.13 [1.68]	1.13 [1.77]	0.98 [1.59]^d^
Year 6	1.16 [1.77]	1.13 [1.85]	1.14 [1.84]	1.10 [1.71]
*Total Difficulties*				
Year 3	6.20 [5.62]	5.89 [5.76]	5.51 [5.41]	4.67 [4.79]^c^
Year 6	5.71 [5.63]	4.82 [5.43]^a^	5.13 [5.53]	4.51 [4.98]^d^
*Numbers rated*				
Year 3	1261	351	652	1593
Year 6	1432	424	1033	1718

*The higher the prosocial score the better the behavior, but for all other scales the higher the score the worse the behavior. Comparison of columns 1 and 2: ^*a*^*P* ≤ 0.01; ^*b*^*P* < 0.05. Comparison of columns 3 and 4: ^*c*^*P* ≤ 0.01; ^*d*^*P* < 0.05.*

### Associations of Child Behaviors Among Women Who Had an External LOC in Pregnancy

Altogether there were 1,612 women who were externally oriented during pregnancy and for whose offspring the teachers’ behavior rating was available at year 3 (1,856 at year 6). As can be seen from **Table 1**, scores for hyperactivity, conduct problems, and total difficulties were significantly better for children of mothers who became internal, but only for year 6; prosocial behavior was unaffected by the change in the mothers’ LOC, but emotional and peer difficulties showed no significant differences.

Comparison of the relationships between the two sexes separately (**Supplementary Tables 1, 2**) shows that, as the external mothers’ orientation changed from external to internal, the behavior of the boys was significantly less hyperactive and they had fewer conduct problems in year 6. No such findings were found in year 3, or for other behaviors in year 6. For girls whose mothers had become internal, all behaviors were better and the differences in prosocial, hyperactive and emotional behaviors as well as total difficulties were statistically better in year 6.

### Associations of Child Behaviors Among Women Who Had an Internal LOC in Pregnancy

The numbers of women who were internally oriented in pregnancy, and whose offspring had been given an SDQ score by their teachers were 2245 and 2751 for years 3 and 6. A comparison of the children whose mothers became external compared with those whose mothers remained internal (**Table 1**) shows that in year 3 those children with mothers remaining internal were significantly more prosocial, less hyperactive, had fewer conduct problems, peer difficulties and total difficulties. In year 6, the children whose mother had become external were significantly more hyperactive, and had increased levels of emotional problems, conduct problems and total behavioral difficulties.

Examination of the sexes separately (**Supplementary Tables 1, 2**) shows boys had significant increases at both time points in the hyperactive and total difficulties scores if their mothers had changed from internal to external. Conduct problems only significantly increased for boys in year 6. Girls only showed associations in year 3 with hyperactivity, conduct problems and total difficulties – each problem behavior was significantly increased if the mother had become external.

### Comparisons Across all Four Groups

In **Table 1**, a comparison of the pattern of results for children whose mothers remained external with those whose mothers remained internal shows poorer offspring behavior if the mother remained external. Comparison of those who became internal (Ext.Int) with those who became external (Int.Ext), however, reveals some slight differences between the two: the Ext.Int group had children with slightly worse behavior in year 3, but slightly better behavior in year 6 than those whose mothers became external. For boys, these differences are most marked for hyperactivity, conduct problems, and total difficulties. For girls they are also marked for emotional difficulties.

### Changes in Paternal LOC Orientation

It should be noted that the numbers of fathers available for analysis were smaller than the number of mothers, and consequently there was less statistical power. Nevertheless, there were significant associations, some similar and some different to those found for the mothers. Among children whose fathers changed from external to internal (**Table 2**) there were significantly better hyperactive and conduct behaviors at both years 3 and 6; the total difficulties score was also better in year 3. For the children whose fathers changed from internal to external, only one behavior showed a significant difference – those in year 3 were less hyperactive. Subdivision by the child’s sex again showed differences in hyperactivity and conduct problems in boys, but not significantly so in girls (excepting those in year 3 whose fathers changed from external to internal) (**Supplementary Tables 3, 4**).

**Table 2:** Relationship between the mean [*SD*] scores of child’s behavior using the Strengths and Difficulties Questionnaire (SDQ) and the **changes in paternal LOC** between pregnancy and 6 years later.

Outcome and school year	Stayed external	Changed external to internal	Changed internal to external	Stayed internal
*Prosocial*				
Year 3	7.90 [2.27]	8.11 [2.21]	7.84 [2.42]	7.91 [2.35]
Year 6	8.03 [2.37]	8.32 [2.21]	8.21 [2.39]	8.23 [2.15]
*Hyperactivity*				
Year 3	2.59 [2.66]	2.12 [2.40]^a^	2.29 [2.56]	1.96 [2.28]^b^
Year 6	2.23 [2.61]	1.72 [2.41]^a^	1.72 [2.43]	1.56 [2.15]
*Emotional*				
Year 3	1.41 [1.91]	1.40 [1.90]	1.24 [1.94]	1.19 [1.74]
Year 6	1.28 [1.85]	1.23 [1.93]	1.16 [1.79]	1.12 [1.67]
*Conduct Problems*				
Year 3	0.75 [1.56]	0.42 [0.91]^a^	0.59 [1.17]	0.51 [1.13]
Year 6	0.79 [1.52]	0.52 [1.16]^a^	0.58 [1.31]	0.45 [1.06]
*Peer Difficulties*				
Year 3	1.13 [1.67]	1.04 [1.64]	0.98 [1.60]	1.07 [1.70]
Year 6	1.08 [1.73]	1.27 [2.02]	1.06 [1.69]	1.04 [1.63]
*Total Difficulties*				
Year 3	5.89 [5.62]	4.98 [4.88]^a^	5.11 [5.23]	4.73 [4.81]
Year 6	5.39 [5.55]	4.73 [5.65]	4.51 [5.40]	4.17 [4.54]
*Numbers rated*				
Year 3	499	252	288	953
Year 6	593	263	319	1036

*The higher the prosocial score the better the behavior, but for all other scales the higher the score the worse the behavior. Comparison of columns 1 and 2: ^*a*^*P* < 0.05. Comparison of columns 3 and 4: ^*b*^*P* < 0.05.*

## Discussion

In this paper, we have shown that higher numbers of children’s difficulties were associated with higher parent externality, whether externality was unchanged over 6 years or the result of a change toward externality during that time. This was consistent with our past results, showing that children of external parents had more problem behaviors beginning at a very young age (Nowicki et al., 2017, 2018b). We also showed that in no instance did children of parents who remained external or became external over the 6 years have fewer difficulties than children of parents who stayed internal or became internal.

Parent externality has been associated with an assortment of children’s difficulties over the first 11 years of life (Nowicki et al., 2017, 2018b). A core finding from past studies was that the greater the presence of maternal and paternal prenatal externality the greater the problems children experienced. When parental prenatal LOC was mixed, children had fewer problems than when both parents were external, but more difficulties than when both parents were internal. In addition, the parent LOC/child difficulties relation was, at times, differentially affected by each parent’s LOC and whether the child was male or female. In this study, specific associations differed depending on (1) the direction of parental LOC change, (2) which parent changed and in which LOC direction, (3) the sex of the child, and (4) the type of child behavior difficulty shown.

For example, compared to mothers who remained external, mothers’ changing from external to internal had daughters with fewer *total, emotional*, and *hyperactivity* difficulties and more *positive prosocial* ratings. Fathers’ externality did not play as important a role in associations with their daughters as did mothers; compared to fathers who remained external, those who became internal had daughters with fewer difficulties only in *hyperactivity*.

Changes in mothers’ LOC toward internality were more often associated with positive aspects of their daughters’ behavior than fathers’ changes. It may be that mothers’ internality provide a protective buffer for their daughters as they deal with the social demands of preadolescence. Preadolescence is an important developmental period, one in which girls begin to develop more depressive symptomatology than boys (Nolen-Hoeksema, 2002). While there is conjecture as to why preadolescent and adolescent girls develop depression, an association between mothers’ externality and the number of emotion problems involving depression and anxiety may deserve consideration for possibly clarifying the process of why some girls develop depression and others do not. We do know that adolescents’ own internality can act as a buffer against depression in high-risk adolescents (Culpin et al., 2015) and it could be that mothers’ internality functions in the same way for their daughters.

With the exception of *hyperactivity* in year 3, fathers’ change in LOC appears to have little or no relation to their daughters’ difficulties, but that is not the case with their sons. Fathers who became internal as opposed to those who remained external, had sons with fewer difficulties in *conduct* and *hyperactivity* at both School Years 3 and 6. Mothers who became internal had a similar relation to their sons’ outcomes, but only at School Year 6.

The parent internality/fewer conduct difficulties association may help clarify the mechanisms underlying the increase in “acting out” behavior that takes place in preadolescent and early adolescent boys (e.g., Farrington, 2009). Boys who act out are more likely to have parents who show “…inconsistent discipline and low monitoring” (Berg, 2012, p. 634). Mothers and fathers who are internal are more likely to parent with more consistency and control and provide protection against developing behavior difficulties (Campis et al., 1986). This possibility awaits research verification.

The internal to external findings are the mirror opposite to those from external to internal transformations. Parents who became external tended to have children with more difficulties than those who remained internal; parents who became internal tended to have children with fewer difficulties than those who remained external. The presence of externality is associated with more difficulties while the presence of internality reflected fewer problems. The interplay of types of LOC and child difficulties is consistent.

In this paper, we have documented the patterns of change in LOC and child behavior that occur over time in this unique dataset. We have not undertaken analyses to determine the possible mediating factors that may have been a consequence of these changes in LOC, at this stage, but we do intend to do so in the future. For example, we have previously shown that factors such as maternal diet and smoking in pregnancy, whether she breast fed her offspring, read stories or fed the toddler with “junk” food, partly mediated the association between parental externality and the relatively low IQ of the offspring (Golding et al., 2017).

A substantial number of parents changed their LOC orientation over the first 6 years of their children’s life and these changes were differentially associated with children’s difficulties. While we do not yet completely know which life events correspond to parent LOC changes, one study found greater stress involving relationships, finances, and health and illness to be associated with changes toward externality or maintaining externality (Nowicki et al., 2018a). In contrast, less stress and progress toward financial, relational, and healthful stability were associated with remaining internal or becoming internal. Programs to change the identified risk factors associated with externality could be implemented to examine whether the change plays a significant role in changing parent LOC toward internality, and in turn whether changes toward parental internality are associated with positive changes in children’s behavior. In one of the few studies to provide information in this regard, Moreland et al. (2016) examined outcomes in preschool children as a function of parenting LOC (as measured by the specific parental control subscale of the Parental LOC scale) in both mothers and fathers. The authors were interested in determining if a parenting intervention would have a beneficial impact on both the stress level and LOC of parents along with concomitant positive change in their children’s behavior. Before the intervention, externality in mothers and fathers was related to greater disruptive behavior and lower cognitive coping in children. However, the successful intervention not only lowered parental stress and made them more internal, but children’s disruptive behavior decreased, and their coping skills increased suggesting interventions to make parents more internal may also increase the likelihood that children’s behavior also will improve.

If programs are not available to change parent LOC, Nowicki et al. (2018a) suggested that school personnel could provide structured experiences to facilitate children learning to become appropriately internal. With appropriate levels of internality, children may be more able to buffer themselves against the possible negative consequences of parental externality. Hopefully future research will provide evidence to evaluate this possibility.

### Strengths and Difficulties of the Project

A major strength is that the children were observed in school for many months by their class teachers, not family members, eliminating the potential for parental bias. Compared to parents, teachers have more opportunity to observe children’s social and behavioral successes and failures as they interact with a variety of peers and adults across academic and social situations at differing times. They also have the advantage of being able to compare the child’s behaviors with those of his/her peers. A second strength is the longitudinal nature of the study, with LOC measures being obtained before the child is born (thus minimizing bias that may be associated with characteristics of the newborn child). Thirdly, the relationship between the change in the LOC of the study fathers compared with the child’s behavior is unique. Fourth, the numbers involved in the study are substantially greater than any similar study.

Our strategy of categorizing parental LOC in relation to the median has the disadvantage that a small change in some score can significantly change an individual from internal to external, and vice versa. However, this is likely to have minimized, rather than exaggerated any differences. The fact that we have not analyzed the data using multivariable techniques may be seen as a fault. This was, however, a deliberate decision. The factors that are normally taken into account (e.g., maternal teenage pregnancy; smoking and alcohol consumption; employment status; and parental education level) are often, in themselves, the consequence of having an external LOC. Therefore, allowing for such factors would have been an over-control. Consequently we have treated this study as a search for pattern. Elsewhere we assess other consequences of parental LOC orientation to the individual child (e.g., Golding et al., 2017).

## Conclusion

Changes in parental LOC are associated with child outcomes over the first 6 years of the child’s life. If parents’ LOC remains internal or changes toward internality over time, then children’s outcomes are more positive than if they remain external or become external. Although parents remaining internal was always found to be associated with more positive outcomes than when both parents remained external, positive child outcomes were sometimes dependent on whether the child was male or female and which parent was internal or external. Additional research is needed to ascertain whether there is a cause and effect relationship between changes in parental LOC and children’s outcomes.

## Author Contributions

JG and SN: conceptualization, funding acquisition, and writing – original draft preparation. YI-C: writing – review and editing, and project management. SG and GE: data curation, formal analysis, and investigation.

## Conflict of Interest Statement

The authors declare that the research was conducted in the absence of any commercial or financial relationships that could be construed as a potential conflict of interest.
