# Supplementary material for: The Pattern of Stability and Change in Parental Locus of Control Over 6 Years and Teacher Ratings of Child Behavior
Source: Front Psychol. 2018 Aug 8;9:1427. doi: 10.3389/fpsyg.2018.01427 (PMC6092674; doi:10.3389/fpsyg.2018.01427)
Supplement: Supplementary file 1 [file Table_1.DOCX]

Supplementary Table 1. Relationship between the mean [SD] scores of **boys**’ behaviour using the Strengths & Difficulties Questionnaire (SDQ) and the changes in **maternal** LOC between pregnancy and 6 years later.

|  | **Stayed**  **External** | **Changed External to Internal** | **Changed Internal to External** | **Stayed**  **Internal** |
| --- | --- | --- | --- | --- |
| *Prosocial* |  |  |  |  |
| Year 3 | 6.94 [2.58] | 7.01 [2.47] | 7.20 [2.57] | 7.34 [2.49] |
| Year 6 | 7.22 [2.60] | 7.30 [2.59] | 7.25 [2.56] | 7.40 [2.46] |
|  |  |  |  |  |
| *Hyperactivity* |  |  |  |  |
| Year 3 | 3.60 [2.95] | 3.36 [2.96] | 3.19 [2.86] | 2.71 [2.66]^d^ |
| Year 6 | 3.27 [2.91] | 2.68 [2.78]^b^ | 2.88 [2.75] | 2.55 [2.68]^d^ |
|  |  |  |  |  |
| *Emotional* |  |  |  |  |
| Year 3 | 1.31 [1.88] | 1.42 [1.96] | 1.25 [1.85] | 1.13 [1.69] |
| Year 6 | 1.29 [1.91] | 1.34 [1.91] | 1.22 [1.82] | 1.14 [1.67] |
|  |  |  |  |  |
| *Conduct problems* |  |  |  |  |
| Year 3 | 1.06 [1.70] | 0.99 [1.66] | 0.84 [1.44] | 0.70 [1.30] |
| Year 6 | 1.27 [1.91] | 0.84 [1.42]^a^ | 0.97 [1.61] | 0.79 [1.40]^d^ |
|  |  |  |  |  |
| *Peer difficulties* |  |  |  |  |
| Year 3 | 1.46 [2.00] | 1.39 [1.83] | 1.27 [1.87] | 1.17 [1.76] |
| Year 6 | 1.30 [1.85] | 1.41 [2.17] | 1.37 [2.04] | 1.29 [1.84] |
|  |  |  |  |  |
| *Total difficulties* |  |  |  |  |
| Year 3 | 7.44 [6.17] | 7.15 [6.14] | 6.55 [5.77] | 5.70 [5.36]^d^ |
| Year 6 | 7.12 [6.13] | 6.27 [6.09] | 6.45 [6.05] | 5.77 [5.51]^d^ |
|  |  |  |  |  |
| *Numbers rated* |  |  |  |  |
| Year 3 | 616 | 192 | 466 | 788 |
| Year 6 | 708 | 230 | 555 | 866 |
|  |  |  |  |  |

M = Maternal report

T = Teacher’s report

Comparison of columns 1 and 2 = ^a^P<0.001 ^b^P<0.05

Comparison of columns 3 and 4 = ^c^P<0.001 ^d^P<0.05

Supplementary Table 2. Relationship between the mean [SD] scores of **girls**’ behaviour using the Strengths & Difficulties Questionnaire (SDQ) and the changes in **maternal** LOC between pregnancy and 6 years later.

|  | **Stayed**  **External** | **Changed External to Internal** | **Changed Internal to External** | **Stayed**  **Internal** |
| --- | --- | --- | --- | --- |
| *Prosocial* |  |  |  |  |
| Year 3 | 8.38 [2.03] | 8.52 [2.04] | 8.51 [1.96] | 8.69 [1.88] |
| Year 6 | 8.67 [1.91] | 9.03 [1.63]^d^ | 8.73 [1.87] | 8.79 [1.79] |
|  |  |  |  |  |
| *Hyperactivity* |  |  |  |  |
| Year 3 | 1.94 [2.10] | 1.79 [2.10] | 1.65 [2.10] | 1.39 [1.73]^d^ |
| Year 6 | 1.52 [2.08] | 1.01 [1.51]^a^ | 1.11 [1.75] | 0.93 [1.53] |
|  |  |  |  |  |
| *Emotional* |  |  |  |  |
| Year 3 | 1.56 [2.05] | 1.33 [2.09] | 1.27 [1.81] | 1.22 [1.82] |
| Year 6 | 1.31 [1.86] | 0.95 [1.68]^b^ | 1.28 [1.86] | 1.10 [1.66] |
|  |  |  |  |  |
| *Conduct problems* |  |  |  |  |
| Year 3 | 0.50 [1.15] | 0.45 [1.09] | 0.38 [0.97] | 0.27 [0.70]^d^ |
| Year 6 | 0.47 [1.01] | 0.34 [0.81] | 0.34 [0.92] | 0.30 [0.87] |
|  |  |  |  |  |
| *Peer difficulties* |  |  |  |  |
| Year 3 | 1.01 [1.59] | 0.82 [1.42] | 0.97 [1.62] | 0.79 [1.39] |
| Year 6 | 1.04 [1.67] | 0.80 [1.31] | 0.86 [1.54] | 0.89 [1.54] |
|  |  |  |  |  |
| *Total difficulties* |  |  |  |  |
| Year 3 | 5.01 [4.76] | 4.38 [4.88] | 4.26 [4.66] | 3.67 [3.91]^d^ |
| Year 6 | 4.34 [4.72] | 3.10 [3.88]^a^ | 3.60 [4.39] | 3.23 [3.97] |
|  |  |  |  |  |
| *Numbers rated* |  |  |  |  |
| Year 3 | 645 | 159 | 386 | 805 |
| Year 6 | 724 | 194 | 478 | 852 |
|  |  |  |  |  |

M = Maternal report

T = Teacher’s report

Comparison of columns 1 and 2 = ^a^P<0.001 ^b^P<0.05

Comparison of columns 3 and 4 = ^c^P<0.001 ^d^P<0.05

Supplementary Table 3. Relationship between the mean [SD] scores of **boys**’ behaviour using the Strengths & Difficulties Questionnaire (SDQ) and the changes in **paternal** LOC between pregnancy and 6 years later.

| **Behaviours** | **Stayed**  **External** | **Changed External to Internal** | **Changed Internal to External** | **Stayed**  **Internal** |
| --- | --- | --- | --- | --- |
| *Prosocial* |  |  |  |  |
| Year 3 | 7.26 [2.49] | 7.74 [2.22] | 7.08 [2.63] | 7.28 [2.49] |
| Year 6 | 7.45 [2.44] | 7.62 [2.47] | 7.40 [2.65] | 7.65 [2.33] |
|  |  |  |  |  |
| *Hyperactivity* |  |  |  |  |
| Year 3 | 3.25 [2.98] | 2.68 [2.39] | 3.09 [2.94] | 2.55 [2.55]^d^ |
| Year 6 | 3.15 [2.94] | 2.27 [2.71]^a^ | 2.53 [2.85] | 2.26 [2.46] |
|  |  |  |  |  |
| *Emotional* |  |  |  |  |
| Year 3 | 1.20 [1.77] | 1.35 [1.93] | 1.11 [1.78] | 1.16 [1.71] |
| Year 6 | 1.19 [1.89] | 1.15 [1.74] | 1.14 [1.77] | 1.11 [1.69] |
|  |  |  |  |  |
| *Conduct problems* |  |  |  |  |
| Year 3 | 0.93 [1.73] | 0.48 [0.94]^a^ | 0.86 [1.37] | 0.70 [1.34] |
| Year 6 | 1.12 [1.77] | 0.70 [1.34]^b^ | 0.92 [1.68] | 0.65 [1.27]^d^ |
|  |  |  |  |  |
| *Peer difficulties* |  |  |  |  |
| Year 3 | 1.34 [1.74] | 1.27 [1.81] | 1.10 [1.67] | 1.23 [1.84] |
| Year 6 | 1.26 [1.79] | 1.47 [2.07] | 1.30 [1.89] | 1.22 [1.79] |
|  |  |  |  |  |
| *Total difficulties* |  |  |  |  |
| Year 3 | 6.72 [6.09] | 5.77 [4.85] | 6.17 [5.75] | 5.64 [5.24] |
| Year 6 | 6.72 [6.00] | 5.58 [5.85] | 5.90 [6.33] | 5.23 [5.12] |
|  |  |  |  |  |
|  |  |  |  |  |
| *Numbers rated* |  |  |  |  |
| Year 3 | 243 | 133 | 150 | 501 |
| Year 6 | 296 | 132 | 159 | 532 |
|  |  |  |  |  |

M = Maternal report

T = Teacher’s report

Comparison of columns 1 and 2 = ^a^P<0.001 ^b^P<0.05

Comparison of columns 3 and 4 = ^c^P<0.001 ^d^P<0.05

Supplementary Table 4. Relationship between the mean [SD] scores of **girls**’ behaviour using the Strengths & Difficulties Questionnaire (SDQ) and the changes in **paternal** LOC between pregnancy and 6 years later.

| **Behaviours** | **Stayed**  **External** | **Changed External to Internal** | **Changed Internal to External** | **Stayed**  **Internal** |
| --- | --- | --- | --- | --- |
| *Prosocial* |  |  |  |  |
| Year 3 | 8.49 [1.86] | 8.52 [2.14] | 8.67 [1.84] | 8.61 [1.96] |
| Year 6 | 8.62 [2.14] | 9.02 [1.66] | 9.02 [1.77] | 8.85 [1.75] |
|  |  |  |  |  |
| *Hyperactivity* |  |  |  |  |
| Year 3 | 1.98 [2.14] | 1.50 [2.27]^d^ | 1.42 [1.70] | 1.31 [1.72] |
| Year 6 | 1.32 [1.83] | 1.17 [1.91] | 0.91 [1.56] | 0.82 [1.42] |
|  |  |  |  |  |
| *Emotional* |  |  |  |  |
| Year 3 | 1.61 [2.01] | 1.45 [2.07] | 1.39 [2.10] | 1.22 [1.77] |
| Year 6 | 1.37 [1.82] | 1.31 [2.12] | 1.17 [1.81] | 1.13 [1.66] |
|  |  |  |  |  |
| *Conduct problems* |  |  |  |  |
| Year 3 | 0.58 [1.37] | 0.36 [0.87] | 0.30 [0.81] | 0.30 [0.78] |
| Year 6 | 0.47 [1.13] | 0.33 [0.91] | 0.24 [0.65] | 0.24 [0.73] |
|  |  |  |  |  |
| *Peer difficulties* |  |  |  |  |
| Year 3 | 0.94 [1.58] | 0.79 [1.38] | 0.84 [1.52] | 0.89 [1.50] |
| Year 6 | 0.90 [1.64] | 1.08 [1.95] | 0.83 [1.43] | 0.86 [1.41] |
|  |  |  |  |  |
| *Total difficulties* |  |  |  |  |
| Year 3 | 5.11 [5.02] | 4.09 [4.79] | 3.96 [4.34] | 3.73 [4.06] |
| Year 6 | 4.07 [4.71] | 3.88 [5.33] | 3.14 [3.83] | 3.05 [3.52] |
|  |  |  |  |  |
|  |  |  |  |  |
| *Numbers rated* |  |  |  |  |
| Year 3 | 257 | 119 | 138 | 452 |
| Year 6 | 297 | 131 | 160 | 504 |

M = Maternal report

T = Teacher’s report

Comparison of columns 1 and 2 = ^a^P<0.001 ^b^P<0.05

Comparison of columns 3 and 4 = ^c^P<0.001 ^d^P<0.05

Supplementary Table 5. Effect sizes of the differences between the group of **women** who stayed internal over time and those who (i) stayed external; (ii) changed from external to internal, and (iii) changed from internal to external (see Table 1 for the details).

| **OUTCOME AND**  **SCHOOL YEAR** | **Stayed external** | **Changed External to Internal** | **Changed Internal to External** |
| --- | --- | --- | --- |
| *Prosocial* |  |  |  |
| Year 3 | -0.15 | -0.15 | -0.10 |
| Year 6 | -0.06 | -0.06 | -0.07 |
|  |  |  |  |
| *Hyperactivity* |  |  |  |
| Year 3 | 0.30 | 0.25 | 0.19 |
| Year 6 | 0.27 | 0.07 | 0.13 |
|  |  |  |  |
| *Emotional* |  |  |  |
| Year 3 | 0.14 | 0.11 | 0.05 |
| Year 6 | 0.11 | 0.02 | 0.08 |
|  |  |  |  |
| *Conduct Problems* |  |  |  |
| Year 3 | 0.27 | 0.25 | 0.14 |
| Year 6 | 0.26 | 0.05 | 0.11 |
|  |  |  |  |
| *Peer Difficulties* |  |  |  |
| Year 3 | 0.16 | 0.09 | 0.09 |
| Year 6 | 0.04 | 0.02 | 0.02 |
|  |  |  |  |
| *Total Difficulties* |  |  |  |
| Year 3 | 0.32 | 0.25 | 0.18 |
| Year 6 | 0.24 | 0.06 | 0.12 |
|  |  |  |  |

Supplementary Table 6. Effect sizes of the differences between the group of **men** who stayed internal over time and those who (i) stayed external; (ii) changed from external to internal, and (iii) changed from internal to external (see Table 2 for other details).

| **OUTCOME AND**  **SCHOOL YEAR** | **Stayed external** | **Changed External to Internal** | **Changed Internal to External** |
| --- | --- | --- | --- |
| *Prosocial* |  |  |  |
| Year 3 | -0.00 | 0.08 | -0.03 |
| Year 6 | -0.09 | 0.04 | -0.01 |
|  |  |  |  |
| *Hyperactivity* |  |  |  |
| Year 3 | 0.28 | 0.07 | 0.15 |
| Year 6 | 0.31 | 0.07 | 0.07 |
|  |  |  |  |
| *Emotional* |  |  |  |
| Year 3 | 0.13 | 0.12 | 0.03 |
| Year 6 | 0.10 | 0.07 | 0.02 |
|  |  |  |  |
| *Conduct Problems* |  |  |  |
| Year 3 | 0.19 | -0.08 | 0.07 |
| Year 6 | 0.32 | 0.07 | 0.12 |
|  |  |  |  |
| *Peer Difficulties* |  |  |  |
| Year 3 | 0.04 | -0.02 | -0.05 |
| Year 6 | 0.02 | 0.14 | 0.01 |
|  |  |  |  |
| *Total Difficulties* |  |  |  |
| Year 3 | 0.24 | 0.05 | 0.08 |
| Year 6 | 0.27 | 0.12 | 0.07 |
|  |  |  |  |
